# 

**Published:** 1884-09

**Authors:** 


### CONTENTS.

### COMMUNICATIONS.

A Resume of Treatment of Alveolar Abscess.......... 421
A Plea for Education.......... 431
An Old Vulcanite Plate.......... 433
A Bogus Directory.......... 434

PROCEEDINGS.

Meeting of College Faculties.......... 436
Association of State Boards of Dental Examiners.......... 446

To Disguise Taste of Iodide of Potash.......... 448

SELECTIONS.

Iodoform in Dental Surgery.......... 449
What Chauncey Depew Says.......... 460
Odontological Society of Great Britain.......... 461
Chloride of Sodium in Inflammatory Diseases.......... 463
The Sanitary Condition of To-day.......... 464
Electrical Surgery.......... 464

Salmagundi.......... 465

Editorial.......... 468
Proper Medical Fducation.......... 468
Dental College Association.......... 471
American Dental Association.......... 472
Transactions Annual Meeting Illinois State Dental Society.......... 472
Drowned.......... 472

